# The Emotionally Sensitive Child-Adverse Parenting Experiences-Allostatic (Over)Load (ESCAPE-AL) Model for the Development of Secondary Psychopathic Traits

**DOI:** 10.1007/s10567-023-00455-2

**Published:** 2023-09-21

**Authors:** Eva R. Kimonis

**Affiliations:** Parent-Child Research Clinic, School of Psychology, The University of New South Wales, Sydney, NSW 2052 Australia

**Keywords:** Callous-unemotional, Psychopathy, Subtypes, Secondary, Antisocial behavior, Emotional sensitivity, Adverse childhood experiences, Anxiety, Impulsivity

## Abstract

Understanding and treatment of antisocial behavior have improved through efforts to subtype individuals based on similar risk factors and outcomes. In particular, the presence of psychopathic traits is associated with distinct etiological factors and antisocial behavior that begins early in life, is aggressive, persistent, and less likely to normalize with traditional treatments, relative to individuals low on psychopathy or its childhood precursor, callous-unemotional (CU) traits. However, important distinctions can be made *within* individuals with CU/psychopathic traits according to the presence of elevated anxiety symptoms and/or adverse childhood experiences, known as secondary psychopathy/CU traits. This paper provides a broad and brief overview of theory and empirical literature supporting the existence of secondary psychopathy/CU variants as a distinct subtype of childhood antisocial behavior. It outlines the **E**motionally **S**ensitive **C**hild-**A**dverse **P**arenting **E**xperiences-**A**llostatic (Over)**L**oad (ESCAPE-AL) model for the developmental psychopathology of secondary psychopathic/CU traits and discusses research and theory supporting this perspective. Future research directions for testing this conceptual model and its implications for assessing and treating high-risk individuals with secondary CU/psychopathic traits are discussed.

## Introduction

Developmental models of antisocial behavior have evolved considerably in recent years, driven in large part by a growing recognition of heterogeneity among antisocial individuals. Emerging models assume that individuals with antisocial behavior follow risk pathways that diverge markedly from one another in terms of comorbidity, chronicity, and causal mechanisms (Frick & Viding, 2009; Frick et al., 2014). Understanding and treatment of childhood antisocial behavior was advanced by research on callous-unemotional (CU) traits (i.e., callous-lack of empathy, remorselessness, shallow/deficient affect). This research uncovered that antisocial behavior develops via a different developmental pathway for children with CU traits, which is characterized by distinct risk factors, course, prognosis, and treatment response (Frick et al., 2014). Similar to how literature on adult psychopathy informed this paradigm shift—CU traits are closely related to the deficient affective dimension of adult psychopathy and the affective components of conscience in typically developing individuals (Frick et al., 2014; Kochanska, 1993)—early theoretical writings on secondary psychopathy were the foundation for growing understanding that important distinctions can also be made *within* individuals with elevated CU and other psychopathic traits (i.e., narcissistic deceitfulness and impulsive irresponsibility dimensions).

Secondary psychopathy is characterized by high levels of CU/psychopathic traits and antisocial behavior, presenting alongside high anxiety and a marked history of childhood adversity. Within antisocial populations, secondary CU variants^1^ are relatively less prevalent (4–30%) than primary CU variants (13–41%; Craig et al., 2021b). The reason for distinguishing subtypes or variants of CU/psychopathic traits is to further reduce heterogeneity that will inform the etiology and improve treatment of antisocial individuals. This paper revisits the developmental psychopathology of secondary CU/psychopathic traits from a multidisciplinary perspective to offer an updated theoretical model. It focuses on childhood because this is arguably when psychopathology and personality originate. This focus on childhood allows for new perspectives that can build on the literature base to date that largely stems from the downward extension of knowledge about adult psychopathy. This “bottom-up” model aims to advance understanding of developmental pathways to childhood antisocial behavior. This new knowledge is expected to inform the development of mechanistically targeted treatments for children on the secondary CU trajectory. It is intended to be a future-oriented manuscript in its discussion of recommendations for future research directions in the study of secondary CU traits.

## Overview of Psychopathy Subtypes and Variants of CU Traits

Cleckley (1976) originally conceptualized psychopathy as involving a core emotional deficit that is manifested as remorselessness and poverty in major affective reactions; interpersonal features including superficial charm and pathological egocentricity; and antisocial behaviors that are inadequately motivated, and indicative of poor judgment and failure to learn from experience. This conceptualization was later operationalized using empirically derived tools, such as Hare’s (1991) Psychopathy Checklist (PCL/PCL-R) developed within forensic populations. This multidimensional psychopathy construct is composed of theoretically discrete subtypes whose antisocial behavior is thought to emerge via etiological processes that are distinct from one another.

Beyond the well conceptualized and highly researched “primary” (also variously termed “idiopathic,” “essential,” “constitutional,” “anethopathy” Karpman, 1948a; “true,” “simple,” Maughs, 1941; “fundamental” Porter, 1996; and “real” Hicks & Drislane, 2018) psychopathy that aligns with Cleckley’s (1976) case descriptions, a second subtype has been variously termed “symptomatic,” “secondary,” “neurotic” psychopathy (Karpman, 1941, 1948b), a “psychopathic façade” (p. 524, Karpman, 1948b), “acquired callousness” (Kerig & Becker, 2010), and a psychopathy “phenocopy” (p. 530, Mealey, 1995). Of these terms, “secondary psychopathy” has emerged as the preferred terminology across the literature. Most attempts to distinguish between primary and secondary psychopathy focus on either specific dynamics of temperament, different early environmental etiological processes, or both (Karpman, 1941, 1948b; Lykken, 1957; Mealey, 1995; Porter, 1996). This focus on developmental processes involved in secondary psychopathy prompted its initial extension to juvenile populations (Kimonis et al., 2011; for a review see Craig et al., 2021b).

The dominant analytic strategy for uncovering psychopathy and CU variants in contemporary subtyping research involves using various clustering methods (e.g., mixture-modeling), but the range of indicators included in these analyses vary considerably; from general personality traits (Hicks et al., 2004, 2010) to psychopathy facets (Mokros et al., 2015) to a combination of psychopathy/CU factors with indices of anxiety symptoms and/or childhood maltreatment/trauma exposure (Fanti et al., 2013; Kahn et al., 2013; Kimonis et al., 2011). In the child and adolescent literature, operationalizing variants based on anxiety scores is most common (Craig et al., 2021b). Despite differences in data analytic strategy and sample across subtyping research, most studies conducted in the past decade identify at least two high psychopathy or CU variants: a primary variant with low to average anxiety levels and no notable history of childhood adversity, and a secondary variant with pronounced high anxiety levels and a marked history of adverse childhood experiences (Fanti et al., 2013; Kahn et al., 2013; Kimonis et al., 2011). Notably, these primary and secondary variants are often indistinguishable in their levels of CU/psychopathic traits. Yet, contemporary developmental models for CU traits and antisocial behavior do not adequately consider this important heterogeneity (Dadds & Frick, 2019; Waller & Wagner, 2019). Relative to the primary pathway to CU/psychopathic traits that is underpinned by a heritable/dispositional affective deficit, the secondary pathway is traditionally thought to be particularly influenced by socio-environmental factors. Growing evidence supports that environmental influences play distinct roles in shaping the risk pathways of individuals with high versus low levels of CU/psychopathic traits (Waller et al., 2013); however, relatively few studies disaggregate individuals with antisocial behavior into primary and secondary CU/psychopathy variants, despite research and early theoretical accounts highlighting its importance.

## Theoretical Accounts of Secondary Psychopathy

Benjamin Karpman (1941, 1948b) was the first to propose the existence of two distinct psychopathy subtypes with unique etiologies and phenotypic expressions. Central to Karpman’s (1941, 1948b) operationalization of the secondary subtype was that their psychopathic behavior (a) can be readily attributed to a psychological cause in their environment and (b) is underpinned by neuroses (i.e., anxiety, depression, guilt), relative to the primary subtype. As was common in his time, Karpman provided support for the existence of this secondary subtype using case studies of his patients seen at a large public psychiatric hospital in Washington DC. One patient, Sylvia Budd, began engaging in antisocial behavior in early childhood with symptoms of oppositional defiant disorder (anger/irritability, temper tantrums in kindergarten), conduct disorder (aggression toward peers/animals involving weapons, stealing, school truancy at age 6, property destruction/fire setting), CU (low empathy, unconcerned about performance at school, remorselessness, shallow/deficit affect, punishment insensitivity) and interpersonal (pathological lying, blame externalization) psychopathic traits, and possibly attention-deficit/hyperactivity disorder (ADHD) (inattention, impulsivity), presenting with above average IQ (Karpman, 1941).

Karpman (1941) attributed Sylvia’s psychopathic behavior to her experience of parental rejection, lack of love/care/affection, and neglect by her mother who he described as a promiscuous (likely based on her pre-marital pregnancy) alcoholic. Her mother’s rejection was evidenced in her multiple attempts to “violently abort” Sylvia during the pregnancy, sending her to a day nursery at 5 weeks old where she was physically neglected, followed by a private home then “24-h school” (p. 115), at which she visited Sylvia only 2–3 times per week (Karpman, 1941). The presence of these identifiable environmental insults led Karpman (1941) to classify Sylvia with secondary psychopathy, as opposed to primary psychopathy for which no clear causal and only “constitutional” factors can be identified (p. 527; Karpman, 1948b).

The second key defining feature differentiating subtypes according to Karpman’s typology is neuroses (i.e., anxiety, depression, guilt) in secondary psychopathy. Karpman (1941) did not describe specific anxiety symptoms experienced by case study patients such as Sylvia, but instead conjectured that “neurotic conflicts” (p. 117)–possibly “unrequited love, undischarged hostility, unassuaged guilt, motives of inferiority, [urge for] revenge, frustration” (p. 528)—underpinned Sylvia’s tantrums, rages, and inadequately motivated antisocial and criminal acts (e.g., breaking into a home and burning clothes found inside). Karpman returned to experiences of parental rejection and affection deprivation, particularly those occurring in the absence of other buffers of stress, as being the source of these neurotic symptoms, relative to the internal antisocial motivation of primary psychopaths.^2^ He further speculated that the function of the secondary psychopath’s antisocial acts was to provide “emotional release” (p. 135; Karpman, 1948b) and an “*escape* from emotional situations that the patient found difficult to accept” (Karpman, 1941, p. 136, italics added). In turn, the child’s antisocial behavior elicits increasingly harsh, punitive, and neglectful caregiving, which further fuels the child’s hostility, resulting in a vicious cycle.

The developmental mechanism linking adverse childhood experiences with secondary psychopathy symptoms was elaborated on by Porter (1996) who later reframed secondary psychopathy as a distinctive dissociative disorder. Porter explained secondary psychopathy as akin to posttraumatic stress disorder (PTSD) in involving a “detachment of emotion and cognition/behavior” (p. 179). He theorized that repeated intense adverse and traumatic experiences of parental maltreatment “turn off” or “deactivate” the individual’s otherwise intact developing conscience (1996, p. 183) by producing high levels of trauma-related negative affectivity (i.e., anxiety, depression) and post-traumatic stress responses including dissociation. These symptoms stunt empathic development and produce the pattern of antisocial behaviors and emotional detachment that is core to psychopathy. However, studies have largely failed to find consistent evidence for greater dissociative symptoms among secondary relative to primary CU variants (Poythress et al., 2006; Tatar et al., 2012).

While empirical support for these theoretical accounts of secondary psychopathy varies, there is the most robust support for greater exposure to environmental risk factors in secondary psychopathy relative to primary psychopathy (for reviews see Craig et al., 2021b; Hicks & Drislane, 2018). However, these accounts fail to explain *why* some individuals respond to this social adversity by developing secondary CU/psychopathy and many others respond in other diverse ways ranging from healthy adaptation to transitionary adjustment problems to psychiatric and physical disorders (Jaffee, 2017). That is, an estimated 10–36% of children experience parental maltreatment (van Ijzendoorn et al., 2020), but only a small minority develops the complex comorbid clinical profile seen in secondary CU variants.

## A Revisited Theoretical Model for the Development of Secondary CU/Psychopathy

This model aims to explain what risk factors and developmental processes contribute to the distinct clinical profile seen in children with secondary CU/psychopathic traits. This profile involves co-occurring externalizing problems, internalizing problems, and CU traits, occurring in the presence of adverse childhood experiences, and often includes peripheral symptoms of ADHD/impulsivity, PTSD, and/or borderline personality disorder (BPD) (Cecil et al., 2018; Kahn et al., 2013; Kimonis et al., 2012b; Lee et al., 2010; Vaughn et al., 2009). With evidence for the existence of primary and secondary CU variants from early childhood (age 3: Ezpeleta et al., 2017; Fanti & Kimonis, 2017; Kaouar et al., 2023), this model is heavily informed by developmental psychology, developmental psychopathology, child clinical psychology, and health psychology (i.e., focused on the effects of toxic stress on the developing child) literatures. The proposed developmental mechanisms are intended to be distinct from both primary CU variants and conduct problems presenting in the absence of CU traits (hereafter referred to as “CP-only”).

### Model Overview

**Fig. 1:**
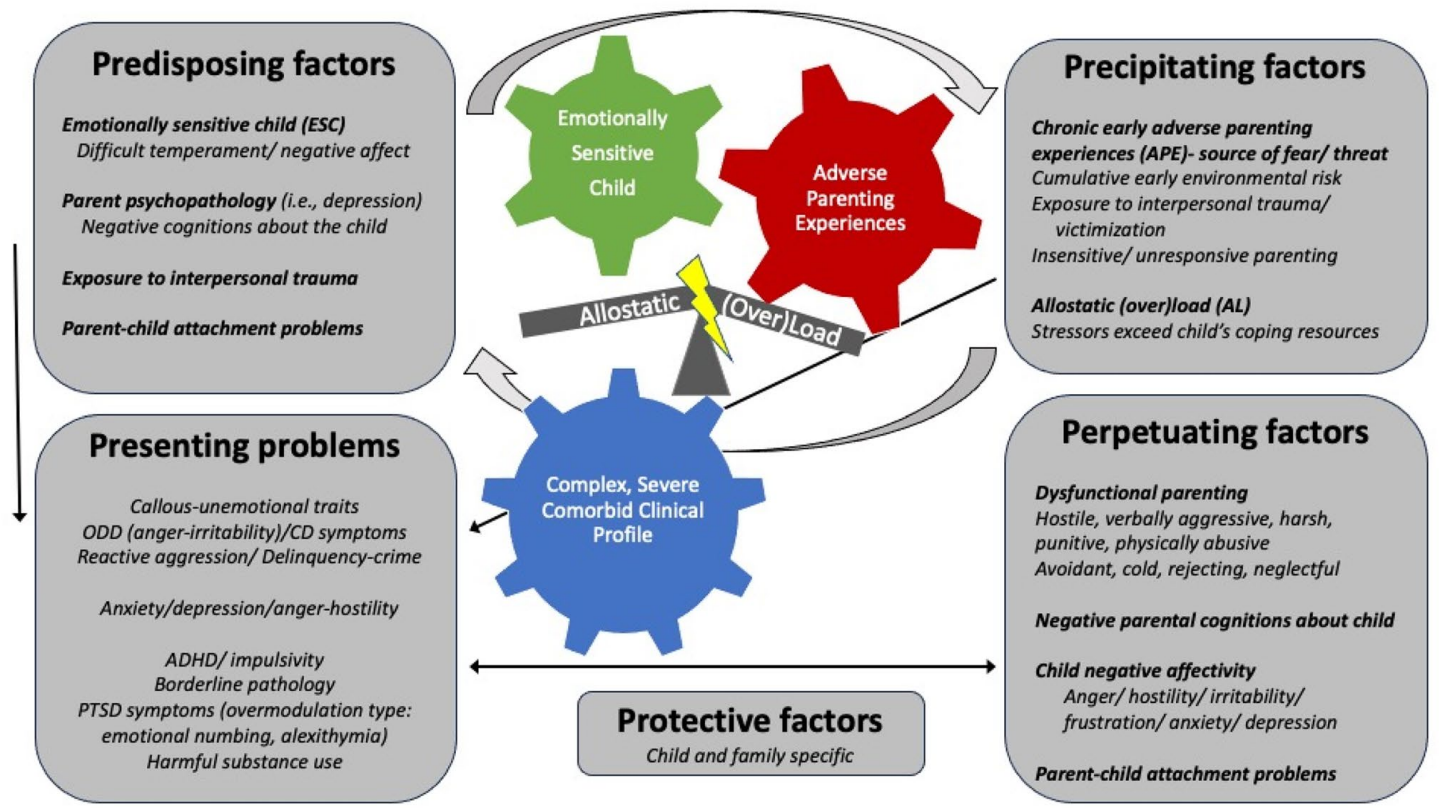
Illustration of the emotionally sensitive child-adverse parenting experiences-allostatic (over) load (ESCAPE-AL) conceptual model of secondary CU/psychopathic traits, embedded within a “Five Ps” clinical formulation model (Kuyken et al., 2008)

This model asserts that the primary mechanisms explaining why individuals who experience prolonged extreme adverse parenting develop secondary CU/psychopathic traits involve: a predisposition to heightened emotion sensitivity in the child that causes them to respond to these experiences with intense emotional and physiological arousal. These responses produce stress levels that are toxic because they are inadequately buffered due to problematic (i.e., disorganized) parent–child attachment and the parent’s failure to provide emotionally sensitive, responsive, and predictable care, and instead being the source of this stress (see Fig. 1). Over repeated increasing aversive parent–child interactions across time, this transactional process damages the child’s developing stress response system, which produces the callous and chronically antisocial, aggressive, and impulsive phenotype that mimics primary CU/psychopathy. The sections below outline the theoretical and empirical bases for this model. They provide a more detailed account of the proposed developmental processes involved in secondary CU/psychopathy and identify future research directions for testing this Emotionally Sensitive Child-Adverse Parenting Experiences-Allostatic (Over)Load (ESCAPE-AL) model.

### Heightened Emotional Sensitivity

The ESCAPE-AL model argues that the extreme levels of anxiety, depression, and anger/hostility (i.e., “neuroses”) seen in individuals with secondary CU/psychopathic traits are underpinned by a dispositional vulnerability involving heightened “emotional sensitivity” to environmental stress. The emotionally sensitive individual has a low threshold for reacting to events in their environment, even those which may be inconsequential to others, with a rapid and extreme emotional reaction from which they are slow to recover. Emotional sensitivity is thought to be evident from birth, manifesting in infancy as hypersensitivity and intense negative affectivity (i.e., excessive crying and difficulty soothing) to environmental stimulation (e.g., novel stimuli such as the presence of unfamiliar adults, others’ emotional expressions, sounds) (Crowell et al., 2009; Linehan, 1993). Models of infant temperament capture this behavioral pattern within dimensions of negative affect/reactivity (i.e., fear, distress to limitations, frustration, anger, soothability; Putnam et al., 2008) and emotionality (i.e., fear, anger, general distress; Buss & Plomin, 1975), which is one of the first in life to emerge and which demonstrates homotypic continuity in predicting distress and negative affectivity at preschool age (Komsi et al., 2006; Putnam et al., 2008; Rothbart, 1989). Temperamental negative affectivity in infancy—particularly involving frustration (i.e., interruption of ongoing tasks/goal blocking) and sadness (i.e., exposure to suffering, disappointment, object loss) subcomponents—predicts co-occurring internalizing and externalizing problems in preschoolers with low effortful control (i.e., emotional and behavioral self-regulation; Gartstein et al., 2012). Similarly, Linehan (1993) explained that the “[emotionally] sensitive child reacts emotionally to even slight frustration or disapproval… annoyance may turn to rage” and “…partings may precipitate very intense and painful grief” (p. 44).

Heterotypic continuity of this heightened emotional sensitivity expresses as difficult temperament in the second year of life and from early childhood as angry-irritable symptoms. Anger-irritability is a subdimension of ODD involving excessive reactivity to negative emotional stimuli, which among other ODD dimensions, uniquely predicts later life internalizing problems (Beachaine & Tackett, 2020; Stringaris, 2011). Extended to adulthood, heightened emotional sensitivity is captured within neuroticism (i.e., the tendency to experience negative affect) from adult personality models (e.g., Costa & McCrae, 1992; Evans & Rothbart, 2007; Eysenck & Eysenck, 1985; Guarino et al., 2007); within the Internalizing (fear, distress subfactors) spectrum of the Hierarchical Taxonomy of Psychopathology (HiTOP, Kotov et al., 2017), and high Acute Threat (“fear”) and Potential Threat (“anxiety”) biobehavioral constructs of the Research Domain Criteria (RDoC, Insel et al., 2010) alternative dimensional frameworks to major classification systems for mental disorders. However, emotional sensitivity is a related, broader construct that is not restricted to threatening stimuli. Rather, adults who suffer from it describe emotional sensitivity as involving frequent, erratic, intense, persistent, and unwanted negative affectivity in reaction to external stimuli, even that which is non-consequential, non-emotional, or unknown to the individual (Wall et al., 2018). Such general situational physiological reactivity (e.g., general corrugator muscle tension) is, however, robustly associated with Acute Threat (i.e., assessed using self-report and multiple physiological indicators of negative emotional reactivity) and anxiety disorder symptoms (Yancey et al., 2016).

There is empirical evidence supporting heightened emotional sensitivity in secondary CU/psychopathy across the lifespan. First, high temperamental negative reactivity to frustration and to novelty at 6-months-old predicted greater mother-reported CU traits at first grade within a highly disadvantaged birth cohort (Wagner et al., 2017). Second, children characterized as secondary CU variants at age 3 were significantly more likely to have a difficult temperament assessed in the first 6 months of life, relative to control children, but not those with primary CU or CP-only (Fanti & Kimonis, 2017). Third, childhood CU traits and internalizing problems are both predicted by earlier levels of angry-irritable ODD symptoms (Barker & Salekin, 2012; Stringaris & Goodman, 2009). Finally, secondary CU variants show heightened attention and arousal to both socioemotional and non-emotional stimuli and heightened sensitivity to threat (i.e., high anxiety or fearful arousal; e.g., Fanti et al., 2018), which contrasts against the reduced pattern that is hypothesized to explain the development of (primary) CU traits in Dadds and Frick’s (2019) Responsiveness, Emotional Attention, and Learning (REAL) model and Waller and Wagner’s (2019) Sensitivity to Threat and Affiliative Reward (STAR) model.

Several studies report that individuals classified as secondary CU/psychopathy variants do not show the same neurobiological and neurocognitive correlates related to the processing of fear and distress stimuli that are found in primary variants (Dadds et al., 2018). For example, incarcerated adolescent boys classified as secondary CU variants showed significantly *greater* attentional orienting to distress cues on a dot-probe task relative to primary CU variants that showed *reduced* attentional orienting to distress (Kimonis et al., 2012a), a pattern that is robustly observed in CU-type conduct problems (e.g., Kimonis et al., 2018). Another study found that incarcerated aggressive male adolescents with secondary CU traits showed *increased* augmented startle reflexes following visual threat prime—a physiological indicator of Acute Threat—compared with those with primary CU traits who showed the characteristic *reduced* augmentation of the startle reflex found in association with psychopathic traits (Kimonis et al., 2017a, 2017b; Patrick et al., 1993). In one of the few fMRI studies, Meffert et al. (2018) found that trauma exposure moderated the association between CU traits and right amygdala responsiveness to fearful facial expressions (see also Sethi et al., 2018). That is, youth with secondary CU traits (high maltreatment) showed *greater* right amygdala reactivity to fear faces relative to those with primary CU traits (low maltreatment) who showed the typical pattern of *reduced* amygdala responses to negative stimuli found in psychopathic youth and adults (Blair, 2013; Marsh et al., 2008; Viding et al., 2012). Another study showed that juvenile variants differed in the functional connectivity of the amygdala (Dugré & Potvin, 2023). These results are consistent with the ESCAPE-AL model in finding that heightened emotional sensitivity is an important risk factor for secondary CU traits.

While patterns of emotional attention and arousal indicative of heightened emotional sensitivity clearly differentiate secondary from primary CU variants, there has been relatively less research comparing CU variants against youth with CP-only for whom heightened emotional arousal in the context of real or perceived threat is central to developmental models (Dodge, 2009; Lochman et al., 2000). The few notable studies conducted to date compared variant groups to incarcerated adolescents scoring low on psychopathic traits, finding some support for greater emotional sensitivity in secondary CU variants relative to CP-only youth. First, Kimonis et al., (2017a) found that secondary CU variants showed enhanced startle potentiation to aversive images relative to youth with CP-only, both those with a history of maltreatment (Cohen’s *d* = 0.65, *p* = 0.06) and without (*d* = 0.58, *p* < 0.05). Second, in a different incarcerated sample Kimonis et al., (2012a) found that juvenile secondary psychopathic variants showed greater facilitation to distress stimuli on a dot-probe task relative to boys low on psychopathic traits, although this moderate effect (*d* = 0.50) did not achieve statistical significance. Thus, relative to youth with CP-only, secondary CU variants are both more emotionally sensitive and show a callous phenotype.

This callous phenotype also distinguishes secondary CU traits from BPD, a construct for which emotional sensitivity is central to its major developmental models (i.e., Linehan’s biosocial theory: Crowell et al., 2009). There is a long-standing debate over whether BPD is a female phenotypic expression of psychopathy (Blackburn, 1998; Sprague et al., 2012). Several studies find that mixed sex adults and youth classified as secondary CU/psychopathy variants show higher levels of BPD symptoms than primary variants (Falkenbach et al., 2014; Goulter et al., 2017; Skeem et al., 2007). These two populations also share several overlapping characteristics, including high rates of early adverse childhood experiences, including maltreatment, trauma, parent–child attachment problems, and poor quality of care (Lyons-Ruth, 2008; Porter et al., 2020); a complex comorbid clinical profile involving anxiety and mood disorders, pathological anger, PTSD symptoms, substance-related disorders, and disorders of impulse including ADHD (Zanarini et al., 1998); and an attentional bias toward negative and threatening stimuli (Jovev et al., 2012; Kaiser et al., 2016). The literature provides less clarity around characteristics distinguishing between these two clinical populations, highlighting the need for studies comparing youth with secondary CU traits and borderline pathology as an important future research direction.

While their shared characteristics leave open the possibility of etiological overlap between BPD and secondary CU traits, these psychopathologies diverge in several key ways. First, unlike secondary CU traits that can be reliably identified in early childhood, BPD is rarely diagnosed before adulthood despite increasing evidence that it emerges earlier than once thought with adult symptoms evident from late childhood and reliably identified during adolescence (Miller et al., 2008). Second, developmental models of BPD and the ESCAPE-AL model diverge in the specific environmental influences giving rise to psychopathology in a biologically vulnerable child. Major etiological models specify that BPD develops for the emotionally sensitive child within an invalidating early family environment in which caregiver(s) are emotionally “unavailable” and intolerant of the child’s expression of negative emotions, particularly those unlinked to observable events (Linehan, 1993). While such emotionally dysfunctional interactions are more likely to occur within abusive or neglectful contexts, current consensus is that child maltreatment is neither necessary nor sufficient to develop BPD (see Crowell et al., 2009). In contrast, the ESCAPE-AL model argues that repeated transactions between the emotionally sensitive child and caregiver(s) that are a source of threat are central to the etiology of secondary CU/psychopathic traits. While it stands to reason that childhood secondary CU traits may be a risk factor for later BPD, or that emotionally sensitive young children who do not experience sufficiently adverse parenting to precipitate allostatic overload later develop BPD and not secondary CU traits, these are empirical questions that future prospective, longitudinal research is needed to test. The ESCAPE-AL model argues that a third key difference between these clinical groups is that stress-induced physiological damage produces the callous phenotype that distinguishes secondary variants from other forms of psychopathology that share similar symptoms and correlates (i.e., BPD, CP-only).

An interesting paradox is that individuals with secondary CU traits show this classic “cool” and callous phenotype while also displaying heightened emotional sensitivity that is evident in their attentional, physiological, and neurocognitive responses. Theory explains that these individuals learn to emotionally detach, despite having the capacity for a full range of emotional experience, in order to *escape* the pain, distress, and fear associated with trauma exposure (Kerig & Becker, 2010; Porter, 1996). Their callous façade has been described as a type of posttraumatic response that serves a self-preservation function as a form of “survival coping” (p. 17, Ford et al., 2006). This pattern of avoiding, controlling, and repressing unwanted emotions, as in dissociation, involves overmodulation of emotions and is characteristic of one PTSD subtype linked to trauma occurring within close interpersonal relationships, relative to an undermodulation subtype that conversely shows hyperarousal and emotional reactivity (Lanius et al., 2010). Mozley et al. (2018) proposed that posttraumatic overmodulation occurring in association with exposure to interpersonal trauma is a risk factor for the development of CU traits, supported by findings that incarcerated youth with secondary CU traits showed both high rates of interpersonal trauma (*v.* non-interpersonal trauma, e.g., accidents, illness, disaster; Kerig et al., 2012) and greater self-reported emotional numbing (specific to fear and sadness), nonacceptance, and lack of clarity around their own emotional states than primary CU variants (Bennett & Kerig, 2014). Other research reports elevated symptoms of alexithymia in secondary CU variants, indicative of an impaired ability to identify and describe their own emotional experiences despite experiencing emotional arousal in distressing situations (Cecil et al., 2018). In contrast to the perspective that overmodulation symptoms are learned in response to interpersonal trauma, the ESCAPE-AL model argues that the mechanism underpinning them involves extreme, unmitigated, and neurotoxic levels of stress experienced in response to chronic severely adverse early caregiving, which exceed the young dispositionally at-risk child’s coping resources and damages their developing physiological stress response system.

### Extreme Adverse Parenting Experiences

There is robust evidence that secondary CU variants experience extreme levels of adverse childhood experiences and particularly dysfunctional parenting (Craig et al., 2021a). The nature of their adversity is broad, ranging from low maternal warmth/sensitivity (Craig et al., 2021a; Fanti & Kimonis, 2017; Kaouar et al., 2023) and affectionless and rejecting parenting (Karpman, 1941), to parenting that is harsh (Goulter et al., 2017; cf. Bégin et al., 2021; Craig et al., 2021a; Humayun et al., 2014), hostile and verbally aggressive (Craig et al., 2021a), to childhood maltreatment (i.e., sexual or physical abuse or neglect; Bégin et al., 2021; Goulter et al., 2019; Kahn et al., 2013; Kimonis et al., 2011, 2012a, 2017a, 2017b; Porter, 1996), to experiencing interpersonal traumatic events including witnessing intimate partner violence and other violence exposure (Bennett & Kerig, 2014; Docherty et al., 2016; Kahn et al., 2013; Sharf et al., 2014; Tatar et al., 2012), to broader cumulative environmental risk across early childhood (Cecil et al., 2014). For example, secondary CU variants identified at age 3 experienced significantly lower levels of maternal sensitivity between 6- and 24-months old, relative to control children and those with CP-only (Fanti & Kimonis, 2017). Relatedly, meta-analytic research (*k* = 29 studies, *N* = 9894) reported a significant moderate (*r* = *0.2*3) association between childhood maltreatment and CU traits, which was stronger at higher levels of anxiety (Todorov et al., 2023; see also de Ruiter et al., 2022).

The limited longitudinal research conducted in this area finds that adverse parenting increases over time for children with secondary CU traits. For example, longitudinal research uncovered that parents of children with secondary CU traits show increasingly harsh, punitive, and physically abusive punishment, and/or avoidant, cold, rejecting, and emotionally neglectful behaviors over time, relative to primary CU variants (Bégin et al., 2021; Craig et al., 2021b). This escalation in adverse parenting may be driven in part by parents’ maladaptive cognitions. Primary caregiver(s) describe the frequent, persistent, intense, and erratic negative affectivity of children with an emotionally sensitive disposition as producing a feeling of “walking on eggshells” (Linehan, 1993). Similarly, at the University of New South Wales Parent–Child Clinic where we assess and treat many young children with conduct problems and secondary CU traits, parents often describe their child in this way. Parents of these young children with secondary CU traits have more negative cognitions about their relationship with the child that are characterized by low warmth and greater feelings of anger/hostility, resentment, contempt, active avoidance and/or a desire to do harm to their child, relative to parents of primary CU variants and CP-only children (Kaouar et al., 2023; see also Reijman et al., 2016). This finding demonstrates the need for future research investigating more nuanced parenting factors associated with CU variants to advance understanding of their development and inform tailoring of parenting interventions.

Studies examining micro-components of parenting behaviors in early childhood found that maternal fright during pregnancy, disinterest during infant feeding (Mendoza Diaz, 2018), low parent–child emotional closeness (i.e., low eye contact, positive and approving facial/ vocal affect, physical proximity during interactions) and emotion communication were uniquely associated with CU traits (Koh et al., 2023), although these studies did not disaggregate variants. Attachment research suggests that frightened and frightening parenting behaviors may originate from parental trauma exposure, leading to an inability to cope with intense negative emotions displayed by their child and contributing to disorganized attachment (Main & Hesse, 1990a, 1990b). Indeed, parents of children with secondary CU traits show higher rates of intimate partner violence and stress-related psychopathology, which are associated with an increased risk of engaging in hostile, rejecting, detached, unresponsive parenting and maltreatment (Cecil et al., 2014; Cohn et al., 1986; Fanti & Kimonis, 2017; van Ijzendoorn et al., 2020). Another potentially important parental risk factor for engaging in these adverse behaviors and warranting future research investigation is parental psychopathic traits, which are associated with child CU traits generally (Mendoza Diaz et al., 2018).

Repeated severe prolonged childhood maltreatment is a precipitating traumatic event within ICD-11’s Complex PTSD diagnosis (CPTSD). Also, exposure to traumatic interpersonal childhood victimization (e.g., emotional/physical abuse, family and community violence) occurring together with disrupted primary caregiver attachment (e.g., caregiver separation, impairment) is central to Developmental Trauma Disorder (DTD), which was proposed as a new and separate diagnosis to PTSD in DSM-5 but ultimately rejected due to limited empirical evidence. CPTSD and DTD share several symptoms in common with secondary CU traits, including impaired interpersonal empathy (DTD), reactive aggression (DTD), emotional and behavioral dysregulation associated with impaired effortful control (DTD, CPTSD), emotional overmodulation (DTD, CPTSD, e.g., dissociation, emotional numbing), threat-related attentional bias (DTD), and attachment problems (DTD), but are also distinct in their requirement that the individual also meet diagnostic requirements for PTSD (CPTSD) that was found to present in the majority (69%) of DTD cases (Spinazzola et al., 2021). Also, relative to children diagnosed with PTSD alone, those meeting criteria for DTD alone had the highest rates of ODD and those with DTD + PTSD had extensive psychiatric comorbidity (Ford et al., 2022). Critically, impaired prosocial emotions co-occurring with high levels of both externalizing and internalizing disorder symptoms are core to secondary CU traits, none of which are required criteria for diagnoses of DTD or CPTSD. Future research is needed to elucidate whether these are separate diagnostic constructs involving distinct etiological pathways or are manifestations of a more complex underlying form of psychopathology that is rooted in the cumulative physiological effects of exposure to traumatic stress whose source is the young child’s primary caregiver(s).

### Allostatic (Over)load

Chronic adverse parenting experiences repeatedly activate the child’s physiological stress response system, producing cumulative wear-and-tear on the body that is termed “allostatic load” (Danese & McEwen, 2012; McEwen & Stellar, 1993). Relative to CP-only children who also experience elevated dysfunctional parenting practices (i.e., harsh and coercive parenting, Dodge et al., 1990; Patterson et al., 1984), the environmental challenges (i.e., increasing frequent and severe angry/hostile, harsh/punitive, cold/rejecting, insensitive parenting) of children with secondary CU traits (a) are more extreme and prolonged; (b) are experienced with more intense emotional and physiological arousal because of the child’s emotionally sensitive disposition; (c) are inadequately buffered; and (d) exceed the developing young child’s physiological ability to cope (Guidi et al., 2021). A child’s ability to adaptively respond to extremely challenging situations and other changing conditions by maintaining physiological equilibrium is termed “allostasis” (Sterling & Eyer, 1988). Allostasis is facilitated in securely attached parent–child dyads by the warm, sensitive, and responsive caregiver who has a buffering effect on the child’s stress levels (Gunnar, 2017; Hostinar et al., 2014). In contrast, for children with secondary CU traits who show the highest rates of disorganized and avoidant attachment styles, even compared against primary CU variants who were predominately securely attached (Cecil et al., 2018), the primary caregiver(s) both exacerbates their stress levels as a central source of fear and threat and at the same time fails to buffer the harmful effects of the chronic, cumulative, and toxic levels of stress they produce in their emotionally sensitized young child (Carroll et al., 2013; Main & Hesse, 1990a, 1990b; van Ijzendoorn et al., 1999).

When a child’s stress response system is continuously activated and buffering factors are inadequate, as in cases of disorganized parent–child attachment and low maternal responsiveness (Ellis & Del Giudice, 2014; Evans et al., 2007), this can produce an extreme state in which allostasis reaches a tipping point known as “allostatic overload” (Fava et al., 2019; McEwen & Stellar, 1993). Allostatic overload resulting from prolonged early adverse childhood experiences dramatically affects brain development, leading to structural and functional abnormalities in several brain areas (i.e., hippocampus, prefrontal and orbitofrontal cortex, amygdala) and altering the maturation and responsiveness of psychophysiological stress response systems (Danese & McEwen, 2012; Weiss & Wagner, 1998). Chronic stress impairs the ability of the hypothalamic–pituitary–adrenal (HPA) axis, the central component of the stress system, to maintain homeostasis and results in dysregulation that is evident in atypical arousal and stress hormone (e.g., cortisol) levels (Danese & McEwen, 2012). For example, relative to non-maltreated children, maltreated children showed greater variability in their afternoon basal cortisol concentrations, which attenuated over time for those with higher initial levels (Doom et al., 2014; Trickett et al., 2010). This altered stress response system may phenotypically express as the callous, antisocial, disinhibited, and comorbid clinical profile that characterizes individuals with secondary CU/psychopathic traits. Allostatic over/load is the hypothesized mediator of chronic stress on such psychological and physical disorders (Danese & McEwen, 2012), and is measurable in the hormonal outputs of the child’s physiological stress response system (Chen et al., 2012).

There are several biomarkers of this HPA axis dysfunction, one of which is an unbalanced ratio between the stress hormone cortisol and the most abundant steroid in the human body, dehydroepiandrosterone (DHEA), which are co-released under stressful conditions. According to the cortisol-DHEA ratio hypothesis, DHEA has a protective function by helping to return the stress response system to homeostasis. In doing so, it aids in buffering the neurotoxic effects of prolonged cortisol exposure on the HPA axis and hippocampus (Kimonides et al., 1999; Young et al., 2002). A high cortisol-to-DHEA ratio in the context of adverse childhood experiences is an indicator of increased chronic stress, HPA axis dysregulation, and is associated with poor mental and physical health (Goodyer et al., 2001). One study found that cortisol-to-DHEA ratios were higher among incarcerated adolescent offenders (N = 232, *M age* = 16.75, *SD* = 1.15) classified as secondary CU variants, relative to all other antisocial subgroups (Kimonis et al., 2017a, 2017b). In this study, secondary CU variants also self-reported the highest levels of childhood maltreatment, stressful life events, and PTSD symptoms.

Findings for cortisol alone are mixed, likely due to methodological differences between studies. One study found lower morning basal cortisol concentrations in 3-year-old secondary CU variants relative to primary CU variants and controls, but not CP-only children (Fanti & Kimonis, 2017), while another found no differences in afternoon basal concentrations between adolescent antisocial subgroups (Kimonis et al., 2017a, 2017b). A third study found that higher basal cortisol levels in 15-month-olds was associated with more high-intensity negative emotional reactivity during a fear-inducing task relative to CP-only and low CP control groups, and predicted membership to a high CP/high CU group at first grade (Mills-Koonce et al., 2015). Infants in the same dataset with higher cortisol reactivity also showed greater antisocial behavior in first grade when they experienced lower maternal sensitivity, but not infants with lower cortisol reactivity (Wagner et al., 2017). Although the latter studies did not disaggregate primary and secondary CU variants, all children were recruited from a birth cohort in areas characterized by high social adversity (i.e., child poverty). Taken together, these findings provide preliminary support for the role of allostatic overload in the development of secondary CU traits, but require replication in other samples and using multi-system indices (e.g., RDoC’s Sustained Threat [“chronic stress”] construct; Goulter et al., 2023a reviews neuroendocrine and inflammatory correlates of CU traits).

### Associated Characteristics

#### Impulsivity/Disinhibition

When examined from the broader psychopathy construct lens, the empirical findings are clear in demonstrating the importance of dysregulated and disinhibited-impulsive behavior to secondary psychopathic traits. From the perspective of the triarchic model of psychopathy (TriPM; Patrick et al., 2009), which identifies boldness (i.e., arrogant and deceitful interpersonal style facet from PCL measures; Hare, 1991), meanness (i.e., CU traits, PCL deficient affective experience facet), and disinhibition (i.e., PCL impulsive and irresponsible lifestyle facet) as the core dimensions of psychopathy, Hicks and Drislane (2018) asserted that individuals with secondary psychopathy are most strongly characterized by disinhibition, followed by meanness. In contrast, those with Cleckleyan (1976) primary psychopathy were thought to be characterized by roughly equal levels of boldness and disinhibition, and lesser meanness. Consistently, several youth studies find higher levels of impulsivity, disinhibition, and symptoms of ADHD in secondary CU/psychopathic variants relative to primary variants and controls (Kahn et al., 2013; Kimonis et al., 2012a, 2012b; Meehan et al., 2017; Vaughn et al., 2009; in adults see also Hicks & Drislane, 2018); however, not all studies find significant differences between variants in psychopathy-linked impulsivity (i.e., impulsive irresponsibility dimension; Kimonis et al., 2011).

Impulsivity emerges in early childhood and increases risk for a host of psychopathologies, most notably those that commonly co-occur with secondary CU traits, i.e., other externalizing disorders, BPD, and substance use disorders (see Beauchaine & Neuhaus, 2008). Etiological models for several of these impulse control disorders assert that early extreme impulsivity is a predisposing vulnerability for the later development of these disorders and behavioral and emotional dysregulation generally (Beauchaine & Neuhaus, 2008; Crowell et al., 2009). Conversely, the current model does not assert that impulsivity, or the related infant temperamental construct of effortful control, are precursors to the dispositional emotional sensitivity that increases susceptibility to developing secondary CU/psychopathic traits. Although heavily biologically driven, low self-regulatory capacity is also associated with chronic stress and early parent–child attachment problems, which potentially contribute to the dysregulated and reactively aggressive behaviors that are commonly observed in maltreated children (Lavi et al., 2019), as well as in individuals with secondary CU/psychopathic traits (Cecil et al., 2018; Craig et al., 2021a; Fanti & Kimonis, 2017; Goulter et al., 2017). Where impulsivity fits temporally in the developmental psychopathology sequence culminating in secondary CU is a question that only prospective, longitudinal research beginning early in life can address.

It is important to acknowledge that impulsivity has been identified as a key factor distinguishing children and adolescents with CP-only from those with CP co-occurring with CU traits (Frick et al., 2014). The literature suggests that, relative to individuals with CP-only, secondary CU variants show more extreme levels of impulsivity/disinhibition (Bégin et al., 2021; Hicks et al., 2004; Kahn et al., 2013; Kimonis et al., 2012a, 2012b). This greater severity is evident in the significantly impaired and comorbid clinical profile of secondary CU variants relative to the CP-only subgroup. This profile is characterized by greater symptoms of conduct disorders, internalizing disorders, ADHD/impulsivity and other psychopathic traits, and poorer cognitive abilities, presenting within a more adverse multisystemic relational context that is characterized by less warm, more hostile, neglectful, rejecting parenting, with further evidence for conflicted student–teacher relationships and peer relationships characterized by greater victimization experiences occurring downstream (Bégin et al., 2021). The ESCAPE-AL model proposes that secondary CU/psychopathic traits is a third and more severe subtype of childhood conduct disorders that develops though a unique etiological pathway relative to CP-only and primary CU variant antisocial subgroups.

When identifying juvenile variant groups using a multidimensional psychopathy approach, it will be important for future research to establish whether measures of psychopathic disinhibition (impulsive-irresponsible lifestyle) and boldness (arrogant-deceitful interpersonal style) add incremental utility beyond well-established child clinical disorders in distinguishing secondary variants from other antisocial subgroups (Salekin, 2022). This is an important consideration because the symptoms comprising disinhibition and boldness overlap with ADHD (i.e., impulsivity) and CD (i.e., deceitfulness/theft) symptoms, respectively (Frick, 2022a, 2022b). In contrast, CU traits (i.e., meanness) are distinct from existing childhood mental disorders, enabling them to be added as a “limited prosocial emotions” specifier to conduct disorders in the most recent revisions of diagnostic classification systems (DSM-5-TR, APA, 2022; ICD-11, World Health Organization, 2019). However, since these classification systems only include specifiers for limited prosocial emotions (DSM-5 CD and ICD-11 ODD and CD) and chronic irritability-anger (ICD-11 ODD), it is important to increase awareness about this additional heterogeneity in childhood externalizing problems and the clinical utility of identifying secondary CU variants.

## Additional Future Research Directions

This final section focuses on further directions for future research aimed at testing this etiological model. Before this discussion, it is important to consider some methodological issues in the future study of secondary CU variants. As discussed above, complex statistical subtyping methods are most often used to identify CU/psychopathy variants within the broad literature base. Within clinical and forensic settings, these methods are likely to be impractical as they rely on advanced statistical knowledge, skills, and software. Instead, it will be necessary to identify simpler, yet reliable methods for classifying CU variants, such as by using clinical cut-offs on common measures of psychopathology and/or adverse childhood experiences. For example, Cecil and colleagues (2018) used a severity-based, cut-off approach to classify high-risk community youths into high- and low-anxious CU variant groups before comparing them on validating measures. Using this approach, their results were largely consistent with prior research in finding greater childhood abuse and neglect, irritability, affective dysregulation, depressive, PTSD, dissociative, alexithymic, and ADHD symptoms, harmful substance use, and suicidal ideation in secondary relative to primary CU variants (see also Sharf et al., 2014). Future research must be undertaken to determine how to best supplement CU/psychopathy measures with measures of psychopathology and social adversity to optimally identify variants. Refining the assessment of secondary psychopathic traits for clinical and forensic contexts will enable the identification of well-characterized clinical groups to whom to administer and test targeted treatments and advance future research into mechanisms underpinning CU variants.

Research into etiologically significant attentional, physiological, psychobiological, neurocognitive, and genomic mechanisms for CU/psychopathic variants is in its infancy. To date, relatively few studies have examined one or more of these factors in samples of children and adolescents and findings from these studies have yet to be replicated in other samples, and against CP-only and control groups to establish deviation from typical developmental processes. Consequently, their robustness for differentiating antisocial subtypes, and whether they play an etiological role or are a consequence of the child’s problem behaviors and/or adverse childhood experiences, is yet to be determined within genetically informed longitudinal studies (Frick et al., 1999). The only genetically informed longitudinal study conducted on CU variants to date found that higher DNA methylation in the oxytocin receptor gene (OXTR) at birth predicted higher CU traits at age 13 at low levels of internalizing problems (primary variant), but not at high levels (secondary variant) (Cecil et al., 2014). Instead, interpersonal pre-natal environmental risk factors (i.e., family conflict, intimate partner violence) were more strongly associated with later CU traits at high internalizing levels (secondary variant) relative to low levels. The authors speculated that pre-natal risk may contribute to secondary CU traits via maternal stress, which is associated with both CU traits (Barker et al., 2011) and internalizing problems (O’Connor et al., 2002).

A necessary future direction for this field is conducting large-scale prospective longitudinal research that incorporates repeated measures of genomic, multi-method environmental and multi-level psychobiological factors, broad-band measures of psychopathology, and multidimensional measures of psychopathic traits to gain a more complete multidomain understanding of the distinct development trajectories of antisocial subtypes (primary and secondary CU variants, CP-only) and low CU/CP control individuals across the lifespan. Regarding epigenetic factors possibly relevant to secondary CU, a lifetime increased vulnerability to heightened stress-reactivity was related to early parental maltreatment via hyper-methylated sites on glucocorticoid receptor gene (exon 1_F_ *NR3C1* promoter region), which affects glucocorticoid receptor gene expression in the hippocampus (McGowan et al., 2009; Meaney & Szyf, 2005). Consistently, clinic-referred antisocial children with hyper-methylated 1_F_ *NR3C1* promoter sites showed increased comorbid internalizing problems and morning cortisol concentrations (Dadds et al., 2015). In order to more fully understand how environment regulates gene expression across development, future research must begin early in life and also examine transgenerational influences, given findings that subsequent generations of normally reared offspring of trauma-exposed animals showed aberrant neural structures and behavioral vulnerabilities via inherited genetic methylation mechanisms (Dias & Ressler, 2014; Franklin et al., 2010).

Important questions for this longitudinal research to address include when subtypes first emerge and can be reliably assessed; whether there are critical points in development when secondary CU/psychopathic traits are most likely to emerge for at-risk individuals; whether non-interpersonal sources of adversity or interpersonal adversity occurring outside of the caregiver-child relationship can precipitate secondary CU traits in a dispositionally vulnerable child; the directionality of influences across development; how stable membership to variant groups is across the lifespan; to what extent individuals phase in and out of these groups; and what factors predict change, such as waxing and waning of anxiety symptoms or experiences of social adversity. The few longitudinal studies conducted to date suggest some developmental stability in variant group membership (Bégin et al., 2021; Goulter et al., 2017), but did not assess individuals past early adolescence. Considering evidence that individuals who experience childhood maltreatment are at increased risk for future victimization in their later relationships (Abajobir et al., 2017), and peer victimization in childhood increased both CU traits and internalizing problems three years later via increasing irritability (Barker & Salekin, 2012), there may be multiple critical points across development at which secondary CU traits onset. Adolescence and puberty, in particular, is a transition period during which normative developmental changes in the neurobiology of stress increases emotional sensitivity to stressors and other emotional stimuli (Dahl, 2004; Quevedo et al., 2009; Spear, 2000). This knowledge is essential to refining understanding of developmental trajectories to CU/psychopathic traits and antisocial behavior. Such large-scale, long-term prospective interdisciplinary research will require significant collaboration and funding. This rests on the scientific community and funding agencies understanding and valuing this line of inquiry.

Another important future direction is to determine whether there are cultural or sex/gender differences in the development and manifestation of primary and secondary CU/psychopathic traits. To date, the majority of CU variants research has been conducted with Western populations that are entirely or predominately male, with some exceptions for sex (e.g., Bennett & Kerig, 2014; Cecil et al., 2018; Craig et al., 2021a; Docherty et al., 2016; Kahn et al., 2013). The few studies comparing variants across sex find that adult female secondary psychopathy variants are more psychologically maladjusted than their male secondary counterparts, as evidenced by their more extreme scores on measures of anxiety, mood instability, and poor resilience to stress (Hicks et al., 2010). However, the literature also appears to support similar developmental processes across gender. For example, similar to findings from exclusively male and mixed-sex samples, in a large (*N* = 1829) exclusively female sample assessed longitudinally from ages 7 to 15, secondary CU variants experienced more harsh parental punishment, greater depressive symptoms, and less self-control at age 7, and had more severe symptoms of CD, depression, and BPD at age 16 than girls classified as primary CU variants and controls (Goulter et al., 2017).

Goulter et al.’s (2017) study and others found that girls are overrepresented within the secondary CU subtype relative to primary CU (e.g., 55% *v.* 31%; Cecil et al., 2018; see also Fanti et al., 2013), consistent with longitudinal findings that environmental factors may be particularly important to the development of stable CU traits among girls (Fontaine et al., 2010; Odgers et al., 2005). Future research is needed to establish what factors explain this overrepresentation of girls within the secondary variant group and whether it is due to their greater susceptibility to emotional sensitivity. That is, within a community pre-school sample Hill et al. (2010) found sex differences in emotional sensitivity, which were enhanced in the presence of recent social adversity (i.e., maternal depression, marital discord). Specifically, girls were more emotionally sensitive than boys, which was positively associated with adversity for girls but negatively associated for boys. Research suggests that this sex difference may be due to differential effects of prenatal maternal stress on the developing child. Higher maternal prenatal cortisol levels were associated with greater infant negative emotionality and fearful temperament, internalizing problems, amygdala hyperreactivity, and greater age 2 cortisol reactivity for girls, but not boys (Braithwaite et al., 2017a, 2017b; Buss et al., 2012; Graham et al., 2019; Ping et al., 2015; Sandman et al., 2013).

Since prenatal cortisol levels are higher among women with anxiety problems (Kane et al., 2014), it is challenging to tease apart whether genetic or environmental influences are at play, or both. Informing this question, in a large twin community sample Humayun et al. (2014) found that the heritability of CU traits did not differ significantly between low-anxious primary and high-anxious secondary CU variants. Both antisocial subtypes were under strong genetic influence and the effects of shared environment were negligible, although this study could not determine whether different genetic factors contributed to the different variant presentations. These findings support the possibility that the source of the emotional sensitivity in secondary psychopathic traits is genetically mediated. Certainly, genetic factors substantially influence the related neuroticism construct (see De Moor et al., 2015). These genetic findings also call into question theoretical models asserting that primary psychopathic traits are genetically based and secondary traits are environmentally determined (Karpman, 1941; Porter, 1996). Indeed, the more contemporary STAR model (Waller & Wagner, 2019) proposes that primary CU traits arise from a heritable predisposition to fearlessness and deficient social affiliation interacting with and shaping a caregiving environment characterized by limited affiliative inputs (i.e., low warmth, high harshness/punitiveness), Although not its focus, the STAR model’s authors appear to suggest that this fearless and low affiliative pattern could develop as an adaptation to extreme environmental pressure (i.e., severe maltreatment, extreme trauma) in secondary CU traits, which they termed “reactionary callousness.” This seemingly exclusive focus on environmental developmental mechanisms contrasts against the ESCAPE-AL model’s emphasis on person-by-context interactions.

## Practical Implications

The original reasons for studying childhood manifestations of psychopathy—to identify a subgroup of antisocial youth with a particularly severe and aggressive pattern of antisocial behavior—are particularly relevant to secondary CU/psychopathic variants that show the highest rates among antisocial individuals of institutional and reactively violent infractions (Kimonis et al., 2011). Relative to primary variants, several studies find that secondary variants show more frequent and severe externalizing problems that are reactive in nature, delinquency, and violent offenses across the lifespan (Ezpeleta et al., 2017; Goulter et al., 2017, 2023b), which are exacerbated by their combination of highly dysregulated behavior and greater substance use problems (Craig et al., 2021b). The Risk-Need-Responsivity model (Bonta & Andrews, 2007) for offender rehabilitation suggests that treatment efforts should focus on secondary CU variants because of their high risk for offending and violence perpetration, by tailoring intervention to their unique needs.

Recent efforts to improve outcomes for children and adolescents with CU traits by tailoring interventions to address their needs, have largely ignored this robust evidence base supporting primary and secondary variants. For example, treatment adaptations for children with CU traits focus heavily on improving children’s emotional skills, based on research showing multilevel emotional deficits to distress in association with (primary) CU traits (Dadds et al., 2012, 2019; Fleming et al., 2022; Kimonis et al., 2019; White et al., 2022). Interestingly, Karpman viewed attempts at treating primary psychopathy as futile since they are “no more trainable than a bear, for he appears to lack the capacity [for conscience]” (1948a, p. 458) … “The true [primary] psychopath is not reachable or amenable to deep psychotherapeutic approach, though, if put in a controlled situation, he can be handled with greater ease.” (1948b, p. 529). Describing one of his patients whom he classified as a primary psychopath, Karpman explained that “Punishment, admonitions, or tender care have had no influence whatever in changing his behavior” (p. 533). In contrast, Karpman (1941, 1948b) viewed secondary psychopaths as “decidedly approachable by psychotherapy” (Karpman, 1948a, 1948b, p. 458) and “a population for which early intervention or treatment in adulthood might be beneficial for society” (Porter, 1996, p. 187).

To date, there is only one known published study on treatment outcomes for CU variants. Fleming et al. (2023) found that 3- to 7-year-old clinic-referred children with antisocial behavior and secondary CU traits showed a faster rate of improvement in parent-reported externalizing and internalizing problems in response to Parent–Child Interaction Therapy adapted for CU traits (PCIT-CU; Fleming et al., 2022; Kimonis et al., 2019); however, their defiant, angry-irritable, and dysregulated (but not aggressive/rule-breaking) behaviors deteriorated from post-treatment to follow-up relative to primary CU variants who maintained their gains. Both groups showed significant improvements in CU traits with an undifferentiated rate of change. While these findings suggest that PCIT-CU may require further personalization for children with secondary CU traits, their larger and more rapid improvements in internalizing and externalizing symptoms over active treatment suggests that the focal attachment-focused PCIT intervention may be beneficial for secondary CU variants for whom disrupted primary caregiver attachment and interpersonal trauma exposure are central. Indeed, it has been argued that standard PCIT’s time-out sequence functions as graded exposure to children’s trauma triggers by providing repeated exposure to safe, calm, and predictable parental limit-setting that extinguishes fear associated with trauma activators such as yelling, emotional abuse, and hitting (Quetsch et al., 2015). With further research evidence supporting the ESCAPE-AL model for secondary CU traits, interventions targeting its identified risk factors can be developed and tested. Critically, research demonstrates that targeting the specific risk factors associated with antisocial subtypes improves their treatment outcomes (Dadds et al., 2012; Fleming et al., 2022).

## Conclusion

This paper provides a broad and brief overview of theory and research on secondary CU traits and psychopathy. This literature highlights the importance of considering variants when researching CU traits and psychopathy. This paper offers the ESCAPE-AL conceptual model for the development of secondary CU traits. This model is informed by multidisciplinary and developmental research and aims to weave a coherent picture of the distinct risk factors, including child dispositional emotional sensitivity interacting with chronic and extreme adverse parenting experiences to alter the child’s psychophysiological stress response system via allostatic overload. This process gives rise to the complex, impaired, and clinically severe profile seen in secondary CU variants involving the hallmark callous phenotype presenting alongside externalizing and internalizing problems, and peripheral symptoms of ADHD, PTSD, and BPD. This paper suggests future research directions for testing the ESCAPE-AL conceptual model toward advancing understanding of the developmental psychopathology of secondary CU traits. This research is reliant on feasible, reliable, and valid measurement of CU variants within clinical and forensic populations, which are likely to yield the most individuals with this distinct clinical presentation. These future advances in assessment and classification will inform clinical practice and enable needed future experimental clinical research focused on tailoring treatment to target the mechanisms underpinning secondary CU/psychopathic traits to reduce their significant societal burden.

## Data Availability

No datasets were generated or analyzed because this work proceeds within a theoretical approach.

## Footnotes

1 The terms “subtypes” and “variants” are used interchangeably throughout this manuscript. The term “subtypes” is typically used for categorical distinctions between groups, whereas the term “variants” is used for dimensional distinctions, both of which are used in the broad subtyping literature.

2 Karpman (1948b) acknowledged that psychopathic behavior can lead to parental rejection for the primary psychopath, but also predates it “by a considerable period” (p. 529).
